# Radio-median cubital / radiocephalic arteriovenous fistula at elbow to prevent vascular steal syndrome associated with brachiocephalic fistula: Review of 320 cases

**DOI:** 10.4103/0970-1591.33721

**Published:** 2007

**Authors:** Anant Kumar, Mrigank S. Jha, Manish Singla, Nitin Gupta, Pamposh Raina, Deepak Dubey, Aneesh Srivastava

**Affiliations:** Department of Urology and Renal Transplantation, Sanjay Gandhi Post Graduate Institute of Medical Sciences, Lucknow - 226 014, UP, India

**Keywords:** Arteriovenous fistula, brachiocephalic, radiocephalic, radiomedian cubital

## Abstract

**Aim:**

Radiocephalic arteriovenous fistula (AVF) at wrist is the vascular access of choice for dialysis. In the absence of a suitable vein at the wrist, a brachiocephalic fistula at elbow is usually constructed. In order to avoid the complication of vascular steal syndrome associated with the brachiocephalic fistula, an alternative operative technique involving the creation of radio-median cubital vein / radiocephalic fistula at elbow was evaluated.

**Settings and Design:**

Retrospective study.

**Materials and Methods:**

Between January 1990 and October 2005, 320 patients underwent creation of radio-median cubital vein / radiocephalic AVF at elbow as a primary procedure or following failure of a fistula at the wrist. A transverse skin incision was made 4cm below the elbow crease, centering in line with the brachial artery pulsation. The median cubital vein / cephalic vein was anastomosed to the radial artery in end to side fashion. The surgical complications and patency of the fistulae were analyzed in the immediate and late postoperative period.

**Results:**

Mean operative time was 55 ± 7.15 min. There were no major intraoperative complications. Immediate patency and a palpable distal radial pulse were present in all the patients. Mean time to fistula maturation was 26 ± 5.2 days. No patient developed a vascular steal syndrome at a median follow-up of 54 months (range 12–168 months) Early fistula failure was seen in 16 (5%) patients whereas eight (2.5%) fistulas failed at a later date. Pseudoaneurysm of the arterialized vein at the fistula site developed in only one (0.3%) patient. Pseudoaneurysm proximal to the anastomosis developed in three (0.9%) patients. Sixteen (5%) patients requested for closure of the fistula following successful renal transplant due to unsightly dilated veins and continuous noisy murmur disturbing their sleep.

**Conclusions:**

The radio-median cubital vein / radiocephalic AV fistula at elbow is safe and is a better vascular access procedure for hemodialysis than brachiocephalic fistula because it leads to the dilatation of both the cephalic and the basilic veins with no incidence of vascular steal phenomenon in our experience. Patency and flow rates are similar to brachiocephalic fistula.

Vascular access is the lifeline of a patient with end-stage chronic renal failure.

Brescia-Cimino radiocephalic arteriovenous fistula (RC-AVF) at the wrist remains the vascular access of choice for dialysis even today.[1] When a RC-AVF is not possible due to poor quality veins or after failure of arteriovenous fistula at wrist, the usual solution is to construct a more proximal fistula, usually a brachiocephalic arteriovenous fistula (BC-AVF).[2] Other options are synthetic graft or transposed basilic vein AVF.[3] However, these three types of vascular access have a few disadvantages: i.e., difficult to construct, more prone to steal syndrome, distal arm swelling and infection.[3,4] The most troublesome complication of brachiocephalic fistula at elbow is vascular steal syndrome, which often necessitates the closure of the fistula.

We describe an alternative operative technique to avoid the vascular steal syndrome, which consists of creation of radio-median cubital vein / radiocephalic fistula at the elbow instead of brachiocephalic fistula.

## MATERIALS AND METHODS

**Figure 1:**
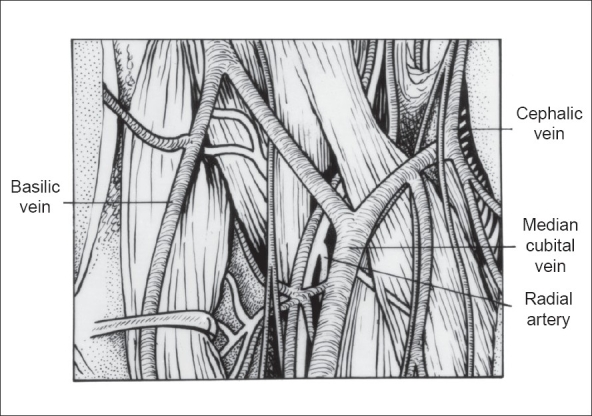
Anatomy of the antecubital fossa

All patients referred for construction of AVF to our department were clinically examined by the operating surgeon for quality of the venous system of the upper limbs. Venous examination included inspection and palpation of the cephalic vein at the wrist and upper arm and the basilic vein at the elbow, after application of a tourniquet in the upper arm. Normal dual blood supply was confirmed by Allen's test. Duplex ultrasound scanning of the veins was performed when physical examination failed to disclose adequate vessels or there was uncertainty regarding the quality or continuity of the vein for arteriovenous access. If a subclavian catheter had been inserted in the past, ultrasonographic assessment of the subclavian vein was mandatory to rule out any stenosis. All patients with poor quality veins at the wrist or patients in whom previous AVF at the wrist had failed were taken up for making a radio-median cubital vein / radiocephalic fistula at the antecubital fossa. [Figure 1]. All patients were taught active arm and hand exercises in the postoperative period to aid fistula maturation. A retrospective review of the data was done for all these patients.

### Operative procedure

We always ensured that the patient was not dehydrated and his blood pressure was within normal limits. Whereas the skin incision in brachiocephalic fistula is a transverse incision in the elbow crease, the incision in radio-median cubital / radiocephalic fistula is about 4cm below the crease, centering over the brachial artery pulsation. Median cubital vein was dissected first and if found suitable, i.e., patent, distensible and of adequate length (> 2.0 cm) for an end to side anastomosis, only then the artery was dissected. If median cubital vein was not suitable, we dissected the cephalic vein in the lateral part of the wound. After disconnection, the vein was distended and flushed with heparinized saline solution. If the saline infusion was without resistance, only then the vein was selected for anastomosis. If veins were not suitable, the procedure was abandoned and artery was not exposed.

**Figure 2:**
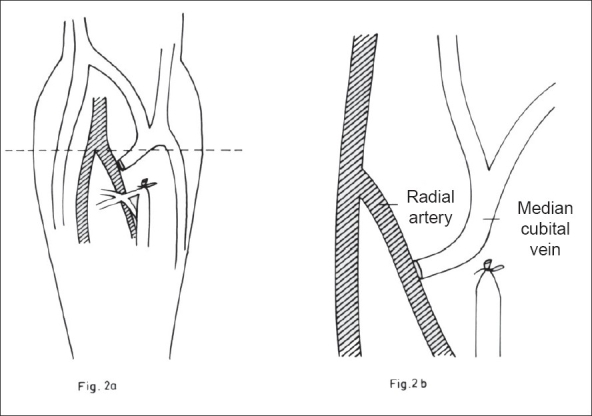
End to side anastomosis of the median cubital vein to the radial artery

The brachial artery was exposed in the median cubital fossa and dissected until it bifurcates into the radial and ulnar arteries. At this junction, the radial artery is still superficial but the ulnar artery enters into deeper compartment. More often, we encounter only the radial artery at this level. Here, the median cubital vein bifurcates and becomes the cephalic and basilic veins. A tributary of this vein often joins the deep vein of the forearm posteriorly. This posterior branch is ligated. The median cubital vein is ligated distally, disconnected, tested for patency and distensibility and then anastomosed to the radial artery in end to side fashion using 6–0 prolene [Figures 2 a and b]. Fistula patency was confirmed on table by presence of a palpable thrill and/or a bruit.

The surgical complications were analyzed in the immediate and late postoperative period. During follow-up, the patency of the fistulas was assessed by either palpation, auscultation or by using a Doppler probe.

Early fistula failure was defined as failure to access the vein for hemodialysis within three months. Delayed fistula failure was defined as those who were successfully accessed initially within three months, but failed at a later date.

The following parameters were studied: age, sex, laterality, comorbid conditions, any previous access failure, mean operative time, presence of distal radial pulse, immediate and late patency rates, the time to utilization for hemodialysis and early and late complications.

## RESULTS

Between January 1990 and October 2005, 320 patients underwent creation of radio-median cubital vein / radiocephalic AVF at elbow as a primary procedure or following failure of a fistula at the wrist. Mean age of the patients was 32 years (range 14–64 years). Majority of the patients were males (4:1) with non-dominant left side being preferable (7:1). Two hundred and seventy-two (85%) patients had a previous failed AVF at the wrist. Eighty (25%) patients had diabetes mellitus as a comorbidity [Table 1]. Patients had a median follow-up of 54 months (range 12–168 months).

**Table 1:** Patient characteristics

Parameters	
No. of patients (n)	320
Age in years (range)	32 (14–64)
Sex (Male: female)	4:1
Previous access failure n (%)	272 (85%)
Diabetes n (%)	80 (25%)
Side (L:R)	7:1

All procedures were performed under local anesthesia. Mean operative time was 55 ± 7.15 min. The median cubital vein could be used for the fistula formation in 260 (81%) patients. The remaining 60 patients in whom the median cubital vein was not suitable in terms of patency, distensibility or adequate length had the cephalic vein utilized for fistula formation. Intraoperative fistula patency was confirmed by the presence of a palpable thrill and/or an audible bruit. There were no major intraoperative complications. Immediate patency and a palpable distal radial pulse were present in all the patients. Mean time to fistula maturation for it to be used for hemodialysis was 26 ± 5.2 days [Table 2]. The cephalic vein was preferred for venous cannulation in 182 (70%) of the 260 patients who underwent a radio-median cubital vein fistula because of its more superficial location and easy accessibility.

**Table 2:** Operative characteristics

Parameters	
Mean operative time (minutes)	55 ± 7.15
Palpable distal radial pulse n (%)	320 (100)
Immediate patency rate n (%)	320 (100)
Early patency rate n (%)	304 (95)
Time to access for hemodialysis (days)	26 ± 5.2

No patient developed a vascular steal syndrome. Early fistula failure was seen in 16 (5%) patients whereas eight (2.5%) fistulas failed at a later date. Pseudoaneurysm of the arterialized vein at the fistula site developed in only one (0.3%) patient. Pseudoaneurysm proximal to the anastomosis following needle trauma during hemodialysis access developed in three (0.9%) patients. Wound infection and thrombophlebitis were noted in 20 (6.3%) and 18 (5.6%) patients respectively. Partial wound dehiscence was seen in seven (2.1%) patients, which required frequent dressings only. No patient had secondary hemorrhage. Sixteen (5%) patients requested for closure of the fistula following successful renal transplant due to unsightly dilated veins and continuous noisy murmur which was disturbing their sleep [Table 3].

**Table 3:** Complications

Parameters	No. (%)
Fistula failure	
Early (< 3 months)	16 (5)
Delayed (> 3 months)	8 (2.5)

Pseudoaneurysm at fistula site	1 (0.3)
Pseudoaneurysm proximal to fistula	3 (0.9)
Request for fistula closure	16 (5)
Wound infection	20 (6.3)
Partial wound dehiscence	7 (2.2)
Thrombophlebitis	18 (5.6)

## DISCUSSION

Ever since the report of the first surgically created arteriovenous fistula for hemodialysis four decades ago, vascular access has remained the Achilles heal of patients on chronic haemodialysis. Arteriovenous fistula using autogenous vein remains the optimal vascular access for hemodialysis. The alternative techniques for hemodialysis, such as long-term central venous catheters and artificial shunts, result in a high rate of infection and thrombosis.[5–7] Brescia-Cimino radiocephalic arteriovenous fistula at the wrist remains the vascular access procedure of choice. However, in patients with poor quality distal veins or with failure of AV fistula at the wrist, other options need to be considered.

Vascular access for chronic hemodialysis should meet the following criteria: long-term patency rate, low complication rate and good acceptance by the patient.

Dixon *et al.*[8] had favored upper arm AV fistulas for higher patency rates. The group had found higher one-, three- and five-year patency rates in upper arm fistulas (71%, 57% and 57%) compared to forearm AV fistulas (54%, 46% and 36%) and grafts (54%, 28%, 0%), but upper arm AV fistulas required more interventions than forearm AV fistulas (1.0 versus 0.6 per access, respectively). Our study compares favorably with the published literature with regards to early and late patency rates (95% and 92.5% respectively), despite 25% of our patients being diabetic. The main causes of primary patency failure were poor condition of the veins and low arterial flow rate. It is important to stress that patients with chronic renal failure should not be dehydrated and/or in hypotension when a fistula is made.

Pseudoaneurysm of the arterialized vein at the fistula site developed in only one (0.3%) patient. This compares favorably with the 2% incidence reported by Zibari *et al.*[9] Pseudoaneurysm proximal to the fistula following needle trauma during hemodialysis access developed in three (0.9%) patients. Pseudoaneurysm at the fistula site required elective resection of the pseudoaneurysm with repair of the radial artery. However, proximal pseudoaneurysm could be successfully repaired in two patients and one patient required closure of the fistula. There was a low incidence of postoperative wound infection (6.3%) and thrombophlebitis (5.6%), which resolved on conservative management in all patients.

Fistula closure was demanded by 16 (5%) patients for dilated tortuous veins. These patients had successful renal transplant and were disturbed by the continuous noise caused by the fistula or had hugely dilated, unsightly veins and opted for the closure of the fistula. The fistula was disconnected and radial artery repaired with 6–0 prolene.

**Figure 3:**
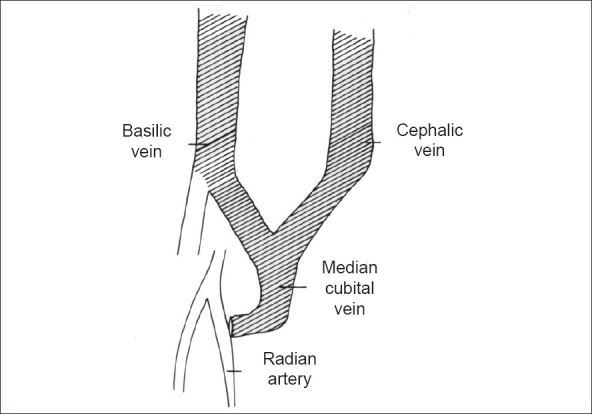
Arterialization of both the cephalic and basilic veins following a radiomedian cubital vein arteriovenous fistula at elbow

Brachiocephalic fistula leads to dilatation of only the cephalic vein, whereas the radio-median cubital vein fistula leads to the dilatation of both the cephalic and basilic veins (through the median cubital vein), thus giving a choice of two arterialized veins in the arm for hemodialysis access [Figure 3]. The time to access for hemodialysis was 26 ± 5.2 days, which is comparable to the existing literature of brachiocephalic and brachiobasilic fistulae wherein only one vein was preserved for the purpose of early maturation. This is a major advantage over the brachiocephalic fistula.

Brachiocephalic fistulae can have high flow rates and hemodynamic complications, such as steal syndrome and high output cardiac failure, occur more commonly than in wrist fistulae. The incidence of vascular steal syndrome is extremely rare following radio-median cubital vein / radiocephalic fistula at the elbow (0% in our series) as against up to 20% with brachiocephalic fistula.[10] The vascular steal phenomenon following brachiocephalic fistula necessitates closure of the fistula. If it occurs in cases of radiocephalic fistula, it can simply be treated by ligating the radial artery distal to the anastomosis. Carpal tunnel syndrome and median nerve compression are also well-recognized complications of brachiocephalic AVF, most cases being secondary to vascular steal syndrome. Radio-median cubital vein / radiocephalic fistula at the elbow is a useful technique to avoid these serious complications as none of the patients in our series of more than 300 cases developed a vascular steal phenomenon. This is due to blood supply by the ulnar artery if the radial artery gets compromised.

This technique is associated with drawbacks, as the incision site is not very cosmetic and has poor healing as compared to the elbow crease incision used for brachiocephalic fistula. However, dilated veins at the elbow or just below it have similar cosmetic significance. Healing at the incision site was excellent in our series with only seven (2.2%) patients having partial wound dehiscence. This procedure also requires a deeper dissection, which is technically more demanding.

## CONCLUSIONS

In the absence of a suitable vein at the wrist, the radio-median cubital / radiocephalic arteriovenous fistula at the elbow is a safe and better vascular access procedure for hemodialysis than brachiocephalic fistula because it leads to the dilatation of both the cephalic and the basilic veins with no incidence of vascular steal phenomenon in our experience. Patency and flow rates are similar to brachiocephalic fistula. It is possible to create this fistula in the majority of the patients with minimal postoperative morbidity and long-term complications.
